# Prion-like propagation of β-amyloid aggregates in the absence of APP overexpression

**DOI:** 10.1186/s40478-018-0529-x

**Published:** 2018-04-03

**Authors:** Alejandro Ruiz-Riquelme, Heather H. C. Lau, Erica Stuart, Adrienn N. Goczi, Zhilan Wang, Gerold Schmitt-Ulms, Joel C. Watts

**Affiliations:** 1 Tanz Centre for Research in Neurodegenerative Diseases, University of Toronto, Krembil Discovery Tower, Rm. 4KD481, 60 Leonard Ave, Toronto, ON M5T 2S8 Canada; 2 Department of Biochemistry, University of Toronto, Toronto, ON M5S 1A8 Canada; 3 Department of Laboratory Medicine and Pathobiology, University of Toronto, Toronto, ON M5S 1A8 Canada

**Keywords:** Aβ, Amyloid, Prion-like transmission, Propagation, Knock-in mice, Cerebral amyloid angiopathy, Amyloid precursor protein

## Abstract

The amyloid cascade hypothesis posits that the initiating event in Alzheimer’s disease (AD) is the aggregation and deposition of the β-amyloid (Aβ) peptide, which is a proteolytic cleavage product of the amyloid precursor protein (APP). Mounting evidence suggests that the formation and spread of prion-like Aβ aggregates during AD may contribute to disease progression. Inoculation of transgenic mice that overexpress APP with pre-formed Aβ aggregates results in the prion-like induction of cerebral Aβ deposition. To determine whether Aβ deposition can also be induced when physiological APP levels are present in the brain, we inoculated *App*^NL-F^ mice, a knock-in model of AD that avoids potential artifacts associated with APP overexpression, with Aβ aggregates derived from the brains of AD patients or transgenic mice. In all cases, induced Aβ deposition was apparent in the corpus callosum, olfactory bulb, and meningeal blood vessels of inoculated mice at 130–150 days post-inoculation, whereas uninoculated and buffer-inoculated animals exhibited minimal or no Aβ deposits at these ages. Interestingly, despite being predominantly composed of protease-resistant Aβ42 aggregates, the induced parenchymal Aβ deposits were largely diffuse and were unreactive to an amyloid-binding dye. These results demonstrate that APP overexpression is not a prerequisite for the prion-like induction of cerebral Aβ deposition. Accordingly, spreading of Aβ deposition may contribute to disease progression in AD patients.

**Electronic supplementary material:**

The online version of this article (10.1186/s40478-018-0529-x) contains supplementary material, which is available to authorized users.

## Introduction

The incidence of Alzheimer’s disease (AD), which is the most common cause of dementia, is expected to triple by the year 2050 [26]. A pathological hallmark of AD is the presence of extracellular amyloid plaques within the brain that are composed of aggregated β-amyloid (Aβ) peptide. Aβ is generated by the sequential cleavage of the amyloid precursor protein (APP) by β- and γ-secretase, producing peptides with lengths that vary from 37 to 43 amino acids [5]. One theory for the origin of AD is the amyloid cascade hypothesis, which proposes that the pathological aggregation and deposition of Aβ in the brain is the initial step in the disease, triggering a series of events that culminate in the aggregation of tau protein into neurofibrillary tangles and, ultimately, neuronal death [54].

One potential explanation for the progressive nature of AD is a continual spreading of protein aggregates within the brain [21, 34]. Indeed, the deposition of Aβ and tau aggregates within the brains of AD patients each follows a stereotypic progression pattern [7, 62]. This is reminiscent of the prion disorders in which self-multiplying (self-propagating) aggregates of the prion protein are responsible for disease spread, both within and between organisms [46]. Accumulating evidence supports a “prion-like” mechanism for the apparent spreading of Aβ pathology during disease [70]. For example, intracerebral or peripheral injection of susceptible transgenic (Tg) mice or rats with brain extracts containing pre-formed Aβ aggregates can induce or “seed” the deposition of cerebral Aβ deposition [18, 19, 38, 41, 50, 69], and Aβ deposits appear to spread away from the inoculation site [17, 72]. Both Aβ aggregates purified from brains and Aβ aggregates formed from synthetic peptides can initiate Aβ deposition when inoculated into transgenic mice, and Aβ-injected animals exhibit electrophysiology and neurogenesis deficits as well as neuronal death [61, 73]. Moreover, like prions, Aβ aggregates can exist as distinct strains with unique conformational properties that produce different pathological phenotypes upon injection into mice [13, 15, 28, 47, 60, 68]. It has also been suggested that a prion-like transmission mechanism may explain the presence of cerebral Aβ pathology in individuals that have received human growth hormone treatments, dura mater grafts, or have undergone neurosurgery [10, 16, 20, 23, 30, 32, 33, 49].

To date, all experimental Aβ propagation studies in rodents have been carried out using models that produce high levels of Aβ via overexpression of wild-type or mutant human APP [22, 35, 41–43, 50]. Although the induction of Aβ pathology is likely governed primarily by the structure and concentration of the Aβ aggregate seeds [41, 42], it has not yet been investigated whether overexpression of APP or the overproduction of one of its non-Aβ cleavage fragments may also contribute to the observed induction and spreading of Aβ deposition. For instance, APP has been reported to act as a receptor for Aβ [36, 40, 55, 58], potentially providing a scaffold upon which Aβ may aggregate. Recently, a homozygous knock-in mouse model of AD was developed in which the mouse *App* gene was replaced with a version containing a humanized Aβ region as well as the Swedish (“NL”) and Iberian (“F”) *APP* mutations [51]. These *App*^NL-F/NL-F^ mice, hereafter referred to as *App*^NL-F^, overproduce human Aβ42 while expressing APP at physiological levels and with the correct spatiotemporal pattern within the brain. Experiments performed using *App*^NL-F^ mice have provided evidence that several phenotypes observed in APP-overexpressing transgenic mouse models of AD may actually stem from APP overexpression rather than Aβ aggregation [51–53]. Here, we demonstrate that Aβ deposition can be induced in the brains of *App*^NL-F^ mice by intracerebral inoculation with Aβ aggregates isolated from either AD patients or a transgenic mouse model of the disorder, confirming that APP overexpression is not a prerequisite for the prion-like propagation of Aβ aggregation within the brain.

## Materials and methods

### Mice

Homozygous *App*^NL-F/NL-F^ knock-in mice [51], which express a modified murine APP that contains the Swedish (KM670/671NL) and Iberian (I716F) mutations as well as a humanized Aβ region, were maintained on a C57Bl/6 background. These mice were generously provided by Takashi Saito and Takaomi Saido (RIKEN Brain Science Institute). Wild-type C57Bl/6 mice were used as a control for APP expression levels. TgCRND8 transgenic mice [12], which overexpress human APP (695 amino acid isoform) containing the Swedish and Indiana (V717F) mutations, were maintained in a hemizygous state on a mixed C57/C3H background. Mice were maintained on a 12 h light/12 h dark cycle and were given unlimited access to food and water.

### Partial purification of Aβ aggregates

The following samples were used: frontal cortex tissue from a 78-year-old female sporadic AD patient (“AD-1”), frontal cortex tissue from a 69-year-old male sporadic AD patient (“AD-2”), and whole brains from two distinct 10-month-old TgCRND8 mice (“TgCRND8–1” and “TgCRND8–2”). Ten percent (wt/vol) brain homogenates were prepared in calcium- and magnesium-free PBS using a Minilys homogenizer and CK14 soft tissue homogenizing tubes (Bertin Corp.). Nine volumes of brain homogenate were lysed with one volume of 10X detergent buffer [5% (vol/vol) Nonidet P-40, 5% (wt/vol) sodium deoxycholate in PBS] and then incubated on ice for 20 min. Lysates were clarified by centrifugation at 1000×*g* for 5 min at 4 °C. Brain extracts were digested with 50 μg/mL proteinase K (PK) for 1 h at 37 °C and then the reactions stopped by the addition of PMSF to a final concentration of 2 mM. Sarkosyl was added to a final concentration of 2% (vol/vol) and then the samples were ultracentrifuged at 100,000×*g* for 1 h at 4 °C. The pellets were resuspended in inoculum diluent buffer [5% (wt/vol) BSA prepared in sterile PBS] and then sonicated for 5 min. Prior to inoculation into mice, the samples were normalized using inoculum diluent buffer so that each contained approximately the same amount of Aβ aggregates, as judged by immunoblotting. The concentration of partially purified Aβ aggregates in the TgCRND8–1 and TgCRND8–2 samples was determined using an Aβ_1-x_ ELISA kit (IBL International) following treatment with formic acid.

### Full purification of Aβ aggregates

Aβ aggregates were purified from the brain of an 11-month-old male TgCRND8 mouse essentially as described previously [61]. The brain was homogenized in 1.5 mL of PBS as described above using the Minilys homogenizer, diluted 1:1 with 2× citrate lysis buffer [20 mM sodium citrate, pH 6; 2% (wt/vol) Triton X-100; 274 mM NaCl; 2 mM EDTA], and then incubated 30 min on ice. 60% (wt/vol) iodixanol (“OptiPrep Density Gradient Medium”; Sigma-Aldrich) was added to the brain homogenate to a final concentration of 18%. Duplicate gradients of 18 (containing brain homogenate), 30, and 35% (wt/vol) iodixanol (top to bottom) were prepared and ultracentrifuged in a MLS-50 rotor (Beckman-Coulter) at 60,000×*g* for 20 min (brake set at level 8). The top layer was discarded and the next two layers were collected, mixed and diluted 1:1 with citrate buffer (10 mM sodium citrate, pH 6; 137 mM NaCl; 1 mM EDTA). This sample was then layered on top of a new gradient containing 25 and 35% (wt/vol) iodixanol (layered with higher concentration at bottom), and then ultracentrifuged at 60,000×*g* for 40 min (brake set at level 8). The top layer was discarded and the next two layers were collected, mixed and diluted 1:1 with citrate buffer. This sample was divided in 1 mL aliquots in siliconized microcentrifuge tubes and centrifuged at 21,000×*g* for 40 min. Pellets were resuspended in 400 μL Tris buffer (10 mM Tris-HCl, pH 8.3; 1.71 M NaCl; 1% (wt/vol) zwittergent 3–14; 1 mM EDTA; 1 mM DTT) and centrifuged at 21,000×*g* for 30 min. The pellets were then resuspended in 50 μL TMS buffer (50 mM Tris-HCl, pH 7.8; 100 mM NaCl; 10 mM MgCl_2_) and combined in one tube. This sample was treated with 150 units of benzonase (EMD Millipore) for 2 h at 37 °C with shaking (600 rpm), divided into two tubes, and centrifuged at 21,000×*g* for 30 min. Supernatants were then discarded and pellets resuspended in 100 μL TMS buffer and combined into one tube. This sample was treated with 20 μg/mL PK for 1 h at 37 °C with shaking (600 rpm). Digestion was stopped with 1 mM PMSF and the sample was adjusted to 1.71 M NaCl, loaded on top of 100 μL of 1 M sucrose, and centrifuged at 21,000×*g* for 30 min. The pellet was then resuspended in 100 μL of 0.1 M ammonium acetate and centrifuged at 21,000×*g* for 30 min. The final pellet was resuspended in 100 μL of dH_2_O. All centrifugation steps were carried out at 4 °C. The concentration of the purified Aβ aggregates was determined using the Aβ_1-x_ ELISA kit following treatment with formic acid. Prior to inoculation, the purified Aβ aggregates were sonicated for 10 min using a water bath sonicator and then diluted 1:10 (vol/vol) in inoculum diluent buffer.

### Intracerebral inoculations

Inoculations were performed as follows: 6-week-old homozygous *App*^NL-F^ mice were anaesthetized using isoflurane gas and then inoculated into the right parietal lobe of the brain with 30 μL of sample at a depth of ~ 3 mm, which corresponds to the hippocampus/thalamus. Each mouse received ~ 0.7 μg of purified Aβ aggregates or ~ 15 ng of partially purified Aβ aggregates. As a negative control, dH_2_O diluted 1:10 (vol/vol) in inoculum diluent buffer was used. Inoculations were performed using a 1 mL tuberculin syringe coupled to a 27-gauge needle (BD #305945). Inoculated mice were monitored daily for routine health. Mice were euthanized at various time points post-inoculation by transcardiac perfusion with saline solution under sodium pentobarbital anaesthesia (50 mg/kg). After perfusion, brains were removed from the skull and divided in half parasagittally. The left half of the brain was frozen and stored at − 80 °C and the right half was fixed in 10% neutral buffered formalin.

### Immunohistochemistry

Formalin-fixed brains were embedded in paraffin and 5 μm sagittal sections were cut and deparaffinized using xylene, 100% ethanol and 95% ethanol, and then processed for immunohistochemistry using standard procedures. Sections to be stained with Aβ antibodies were pre-treated with formic acid for 6 min and blocked using the M.O.M kit (Vector Laboratories). Immunostaining was performed using the following antibodies: anti-Aβ 4G8 (BioLegend; 1:3000 dilution), anti-Aβ40 11A50-B10 (BioLegend; 1:500 dilution), anti-Aβ42 12F4 (BioLegend; 1:2000 dilution), anti-Aβ43 9C4 (BioLegend; 1:500 dilution), anti-oligomeric pE3-Aβ 9D5 (Synaptic Systems; 1:100 dilution), anti-GFAP Z0334 (Dako; 1:500 dilution), and anti-Iba1 (Wako; 1:1000 dilution). Stainings were processed using the ImmPress HRP detection kit (Vector Laboratories), developed using DAB, and counterstained with haematoxylin. To assess amyloid deposition, samples were stained with Thioflavin S [1% (wt/vol) for 8 min], dehydrated using 80 and 95% ethanol, and then mounted using ProLong Diamond Antifade medium containing DAPI (ThermoFisher). Slides were analyzed using a Leica DM6000B microscope and photographed using a 40× objective.

For the quantification of Aβ cerebral amyloid angiopathy, the number of Aβ-positive meningeal blood vessels was expressed as a percentage of the total number of vessels present in the sagittal section. For the quantification of parenchymal Aβ staining, three different fields from the brain region of interest were imaged at 20× magnification and the percentage area covered by Aβ deposition was determined using ImageJ (http://imagej.nih.gov/ij/) and the “IHC analysis toolbox plugin” (http://imagej.nih.gov/ij/plugins/ihc-toolbox/). For each brain region, the values obtained from the three fields were averaged into a single point.

### Brain homogenate preparation and biochemical analysis of mouse brains

Ten percent (wt/vol) brain homogenates were prepared in PBS and then treated with detergent buffer as described above. Lysates were clarified by centrifugation at 1000×*g* for 5 min at 4 °C and then protein concentration in the supernatant was determined using the BCA assay (ThermoFisher). Samples were prepared in 1X Bolt LDS sample buffer containing 2.5% (vol/vol) β-mercaptoethanol, boiled, and then analyzed by immunoblotting.

For analysis of insoluble Aβ, 500 μg of detergent-extracted brain homogenate was treated with a final concentration of 50 μg/mL PK in a volume of 200 μL for a final PK:protein ratio of 1:50. Digestions were performed for 1 h at 37 °C with shaking, and then reactions were halted by addition of PMSF to a final concentration of 2 mM. After the addition of sarkosyl to a final concentration of 2% (vol/vol), samples were ultracentrifuged at 100,000×*g* for 1 h at 4 °C using a TLA-55 rotor (Beckman Coulter). Pellets were resuspended in 1X Bolt LDS sample buffer containing 2.5% (vol/vol) β-mercaptoethanol, boiled, and then analyzed by immunoblotting.

### Determination of Αβ42 levels by ELISA

For quantification of total Aβ42 levels in brain homogenates, protein concentration was determined using the BCA assay and 500 μg of total protein was brought up to 100 μL in PBS. Two volumes of cold 100% formic acid were added to one volume of brain homogenate, followed by sonication in a water bath sonicator for 5 min. Samples were ultracentrifuged at 100,000×*g* in a TLA-55 rotor for 1 h at 4 °C. The resulting supernatants were then dried using a speed vacuum concentrator. Pellets were resuspended in 100 μL of PBS, sonicated for 5 min, and then stored at − 80 °C. For analysis of PK-resistant Aβ42 levels, 500 μg of brain homogenate was digested with a final concentration of 100 μg/mL PK for 1 h at 37 °C with shaking in a volume of 100 μL (diluted with PBS; final PK:protein ratio of 1:50). The digestions were halted by addition of PMSF to a final concentration of 2 mM. Samples were then treated with formic acid and processed identically to the undigested samples. For analysis of soluble Aβ42 levels, brain homogenates were treated with an equal volume of 0.4% (vol/vol) diethylamine/100 mM NaCl, ultracentrifuged at 100,000×*g* for 1 h at 4 °C, and then the supernatants were neutralized by the addition of 0.1 volumes of 0.5 M Tris-HCl pH 6.8. Aβ42 levels were then determined by ELISA (ThermoFisher #KHB3442), which was performed according to the manufacturer’s instructions.

### Immunoblotting and silver staining

Samples were subjected to SDS-PAGE using Bolt 4–12% Bis-Tris Plus gels (ThermoFisher). For separation of individual Aβ isoforms, self-poured Bicine/Tris 10% polyacrylamide gels containing 8 M urea were used [59]. Aβ isoform controls were prepared from recombinant Aβ (rPeptide) and polymerized into PK-resistant fibrils as previously described [60]. Silver stainings were performed using the Thermo Scientific Pierce Silver Stain Kit. For immunoblotting, gels were transferred onto 0.45 μm pore PVDF membranes, blocked with blocking buffer [5% (wt/vol) non-fat skim milk in TBS containing 0.05% (vol/vol) Tween-20 (TBST)], and then incubated with primary antibody overnight at 4 °C. The following antibodies were used: anti-Aβ 6E10 (BioLegend; 1:4000 dilution) or anti-APP 22C11 (EMD Millipore; 1:4000 dilution). Blots were washed 3 times with TBST, incubated with horseradish peroxidase-conjugated secondary antibodies (Bio-Rad) diluted in blocking buffer, and then washed 3 times with TBST. Blots were developed using Western Lightning ECL Pro (PerkinElmer) and exposed to HyBlot CL film. Where appropriate, blots were re-probed with the anti-actin 20–33 antibody (Sigma-Aldrich; 1:10,000 dilution). For quantification of APP levels, densitometry was performed using ImageJ and relative APP levels determined using a concentration curve prepared from serially diluted samples. APP levels were then normalized to the levels of actin present in the sample.

### Electron microscopy

Negative-stain electron microscopy was performed as follows: 9 μL of purified, sonicated TgCRND8 Aβ aggregates were placed on a formvar/copper grid and incubated for 2 min. After removal of sample using filter paper, 9 μL of 1% (wt/vol) phosphotungstic acid (PTA) was dropped onto the grid and incubated for 2 min. Excess PTA was removed and the grid was stored at room temperature in the dark until examined using a Hitachi H7000 TEM microscope.

### Experimental design and statistical analysis

For the experiments involving partially purified Aβ aggregates, 5–9 mice were inoculated per sample. Both male and female mice were used, as indicated in Table 1. For the time-course experiment, 2 female mice per timepoint were analyzed. For the experiments involving fully purified Aβ aggregates, 5 male *App*^NL-F^ mice per sample or timepoint were used. For each age of uninoculated *App*^NL-F^ mice under examination, 2–5 mice were used. Differences in APP levels between wild-type and *App*^NL-F^ mice as well as in Aβ levels between Aβ-inoculated male and female mice were assessed by non-paired, two-tailed *t*-tests. Differences in Aβ42 levels between control- and Aβ-inoculated mice were assessed by one-way ANOVA followed by either Tukey’s or Dunnett’s multiple comparison test. Statistical differences in immunohistochemical staining were analyzed using either non-paired, two-tailed *t*-tests or one-way ANOVA followed by Tukey’s multiple comparison test. In all cases a significance threshold of *P* < 0.05 was utilized. All statistical analyses were performed using GraphPad Prism 5.0.

**Table 1:** Summary of Aβ pathology in inoculated *App*^NL-F^ mice

Inoculum	Days post-inoculation	Age (d)	Sex	Aβ deposition^a^ (n/n_0_^b^)				
				Subcallosal region	Hippocampus	Cortex	Olfactory bulb	Meningeal CAA
Purified TgCRND8 Aβ	14	56	Male	0/5	0/5	0/5	0/5	0/5
	130	172	Male	4/4	0/4	0/4	4/4	4/4
BSA	132	174	Male	0/5	0/4	0/5	0/5	1/5
	151	193	Male	0/5	0/5	1/5	0/5	0/5
			Female	0/4	0/4	1/4	0/4	0/4
TgCRND8–1	30	74	Female	0/2	0/2	0/2	0/2	0/2
	60	104	Female	2/2	0/2	0/2	0/2	1/2
	90	134	Female	2/2	0/2	0/2	1/2	2/2
	150	194	Male	3/3	0/3	1/3	2/3	3/3
			Female	2/2	0/2	0/2	1/2	2/2
TgCRND8–2	152	195	Male	3/4	0/4	0/4	2/4	4/4
			Female	4/4	0/4	2/4	2/4	4/4
AD-1	150	197	Male	3/4	0/4	0/4	3/4	4/4
			Female	3/4	0/4	1/4	1/4	4/4
AD-2	151	195	Male	3/3	0/3	0/3	1/3	3/3
			Female	4/5	0/5	2/5	3/5	5/5

^a^Aβ deposition was assessed by immunohistochemistry using the anti-Aβ antibody 4G8

^b^n, number of positive mice; n_0_, number of analyzed mice; a sample was scored as positive if there was clear evidence for Aβ deposition or at least one Aβ CAA-positive meningeal blood vessel

## Results

### Kinetics of spontaneous Aβ deposition in *App*^NL-F^ mice

The potential for confounding effects of APP overexpression in Tg mouse models of AD led us to investigate the feasibility of using *App*^NL-F^ mice to study the induction and spreading of cerebral Aβ deposition. For all of our experiments, we used homozygous *App*^NL-F^ knock-in mice, which express a partially humanized APP molecule at similar levels to endogenous, wild-type APP in non-transgenic C57Bl/6 mice (Additional file 1: Figure S1). Due to the presence of the Iberian APP mutation, *App*^NL-F^ mice predominantly accumulate the Aβ42 isoform [51]. Thus, to analyze the kinetics of Aβ accumulation in *App*^NL-F^ mice, levels of formic acid-extractable Aβ42 in the brains of male mice at 6, 9, 12, 16, and 20 months of age were determined by ELISA. Cerebral Aβ42 levels gradually increased with age (Additional file 2: Figure S2a) and were similar to what has been previously observed [51]. Insoluble, proteinase K (PK)-resistant Aβ, which is indicative of the presence of aggregated Aβ, was first observable by immunoblotting in brain homogenates from a subset of *App*^NL-F^ mice at 12 months of age, and was present in all mice by 16 months of age (Additional file 2: Figure S2b). Immunohistochemistry with the 4G8 antibody revealed robust cortical Aβ deposition in the brains of *App*^NL-F^ mice by 9 months of age (Additional file 2: Figure S2c, Additional file 3: Table S1). Small and infrequent cortical Aβ deposits (typically 1–2 per section) were also observed in the brains of all 6-month-old female *App*^NL-F^ mice but only rarely in male animals at this age (Additional file 2: Figure S2c, Additional file 3: Table S1). By 12 months of age, Aβ deposits in *App*^NL-F^ mice were also found in the hippocampus and olfactory bulb (Additional file 2: Figure S2c, Additional file 3: Table S1). Accumulation of Aβ within the meningeal blood vessels, indicative of Aβ cerebral amyloid angiopathy (CAA), was readily found in mice of at least 12 months of age (Additional file 2: Figure S2c), but was also infrequently observed in mice at 6 or 9 months of age (Additional file 3: Table S1). Taken together, these results suggest that minimal spontaneous Aβ deposition is present in the brains of *App*^NL-F^ mice before 9 months of age, providing a window during which the induction of cerebral Aβ deposition can be assessed.

### Injection of partially purified Aβ aggregates into *App*^NL-F^ mice

To test whether cerebral Aβ deposition could be induced in *App*^NL-F^ mice, we intracerebrally injected 6-week-old animals with Aβ aggregates isolated from either aged TgCRND8 mice, which rapidly develop cerebral Aβ deposits and accumulate high levels of pathogenic Aβ42 species [12], or sporadic AD patients. To this end, we used a non-stereotactic injection technique in which Aβ aggregates are introduced into the right parietal lobe as an effective means of inducing cerebral Aβ deposition [60, 61, 68]. Two distinct TgCRND8 samples (TgCRND8–1 and − 2) and two unique sporadic AD samples (AD-1 and -2) were injected. As a negative control, mice were inoculated with inoculum diluent (BSA). To enrich for aggregated Aβ species, TgCRND8 and AD brain extracts were partially purified by digestion with PK and then ultracentrifuged prior to inoculation. None of the inoculated mice exhibited any overt signs of neurological illness over the course of the experiment, although one BSA-injected mouse and one mouse inoculated with AD-2 were found dead in their cages at 18 and 94 days post-inoculation (dpi), respectively.

**Fig. 1:**
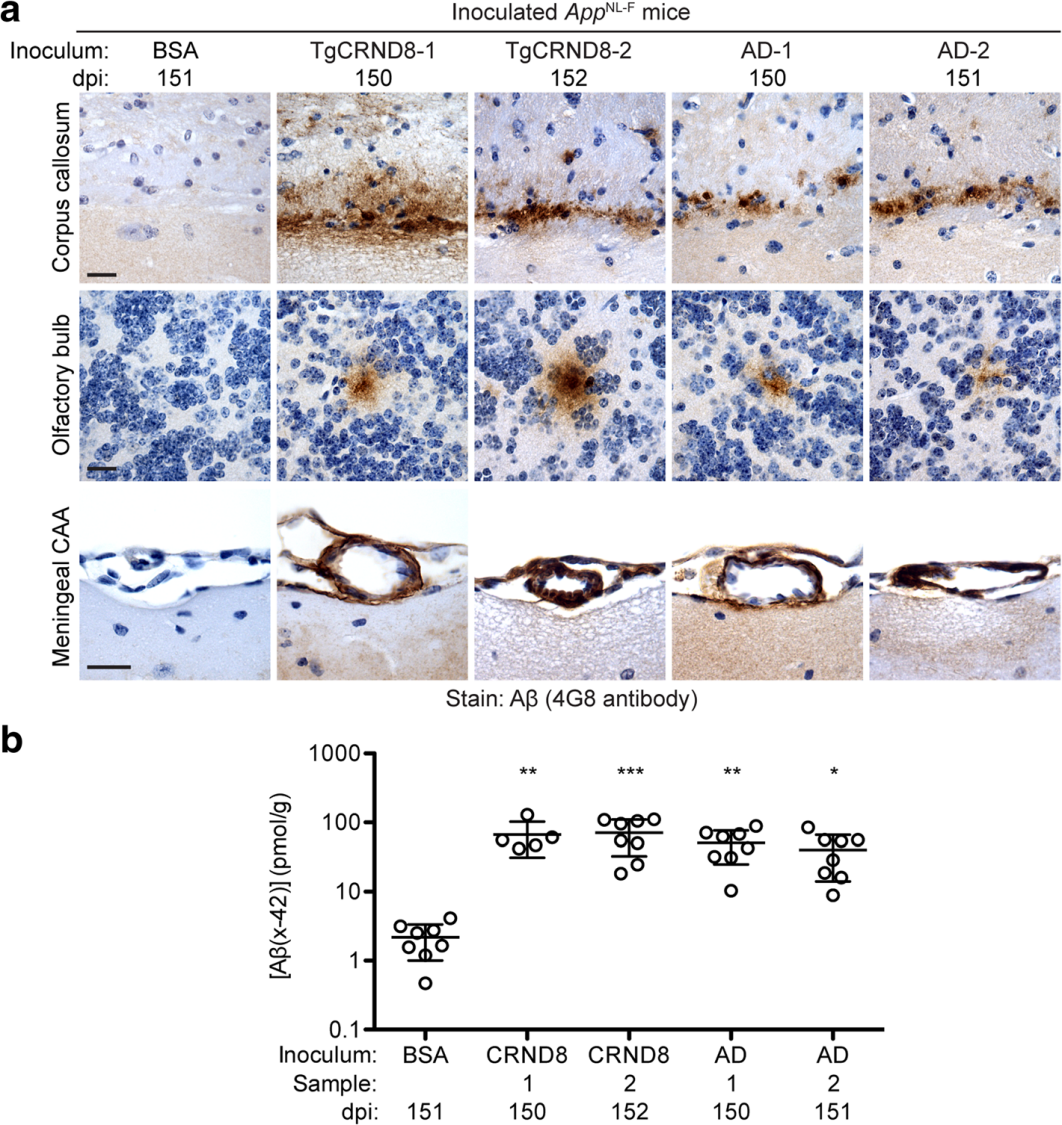
Induction of cerebral Aβ deposition in *App*^NL-F^ mice inoculated with partially purified Aβ aggregates. **a** The brains of male mice inoculated with BSA or partially purified Aβ aggregates from the brains of aged TgCRND8 mice or sporadic AD patients were analyzed by Aβ immunohistochemistry (4G8 antibody) at 150–152 dpi. Representative sections from the corpus callosum, olfactory bulb, and meningeal blood vessels of inoculated mice are shown. Scale bars: 20 μm. **b** Total (formic acid-extractable) Aβ42 levels (mean ± s.d.), as determined by ELISA, in brain homogenates from *App*^NL-F^ mice at 150–152 dpi with partially purified TgCRND8 Aβ aggregates (two samples, *n* = 5 and 8) or partially purified AD Aβ aggregates (two samples, *n* = 8 each) are significantly higher than in mice injected with BSA (n = 8). ***P < 0.001; **P < 0.01; *P < 0.05

The brains of mice inoculated with partially purified Aβ aggregates were analyzed by immunohistochemistry for Aβ deposition at 150–152 dpi. Induced Aβ deposits were apparent in the ipsilateral hemispheres of all of the *App*^NL-F^ mice injected with partially purified Aβ aggregates from TgCRND8 mice or AD patients (Fig. 1a, Table 1). The majority (25/29) of Aβ-inoculated mice exhibited induced Aβ deposits within the subcallosal region located at the interface between the corpus callosum and the oriens layer of the hippocampus, and about half (15/29) of the mice possessed Aβ pathology within the olfactory bulb (Table 1). Aged uninoculated *App*^NL-F^ mice also exhibited callosal Aβ pathology (Additional file 3: Table S1), but the Aβ deposits were located in the corpus callosum itself, distinguishing them from the induced Aβ deposits found in Aβ-inoculated mice (Additional file 4: Figure S3). The induced cerebral Aβ deposits within the subcallosal region and olfactory bulb were primarily diffuse or granular in nature (Fig. 1a). In contrast, none of the BSA-injected mice exhibited any Aβ deposits within these two brain regions (Fig. 1a, Table 1). The deposition of Aβ within the walls of blood vessels in the brain has been previously observed to be associated with the induction of Aβ deposition in transgenic mouse models of AD that overexpress APP [9, 19, 61, 68]. All of the mice injected with partially purified Aβ deposits, but none of the control-injected animals displayed Aβ deposits within the meningeal blood vessels, suggestive of CAA (Fig. 1a, Table 1). A minor fraction (~ 20%) of Aβ-injected mice also exhibited sparse Aβ deposits within the cerebral cortex (Table 1). However, since these deposits were predominantly observed in female mice, which spontaneously develop Aβ pathology more rapidly than males [11], and were also present at a similar frequency in control-injected and uninjected animals (Table 1, Additional file 3: Table S1), the most likely explanation is that they represent spontaneous, non-induced Aβ deposits. Analysis of formic acid-extractable Aβ42 levels in brain homogenates prepared from the contralateral hemisphere of inoculated *App*^NL-F^ mice by ELISA revealed that mice inoculated with partially purified Aβ aggregates accumulated ~ 20–30-fold more Aβ than control-inoculated mice (Fig. 1b). Collectively, these results demonstrate that cerebral Aβ deposits can be induced in *App*^NL-F^ mice by inoculation with partially purified Aβ aggregates from either TgCRND8 mice or AD patients.

**Fig. 2:**
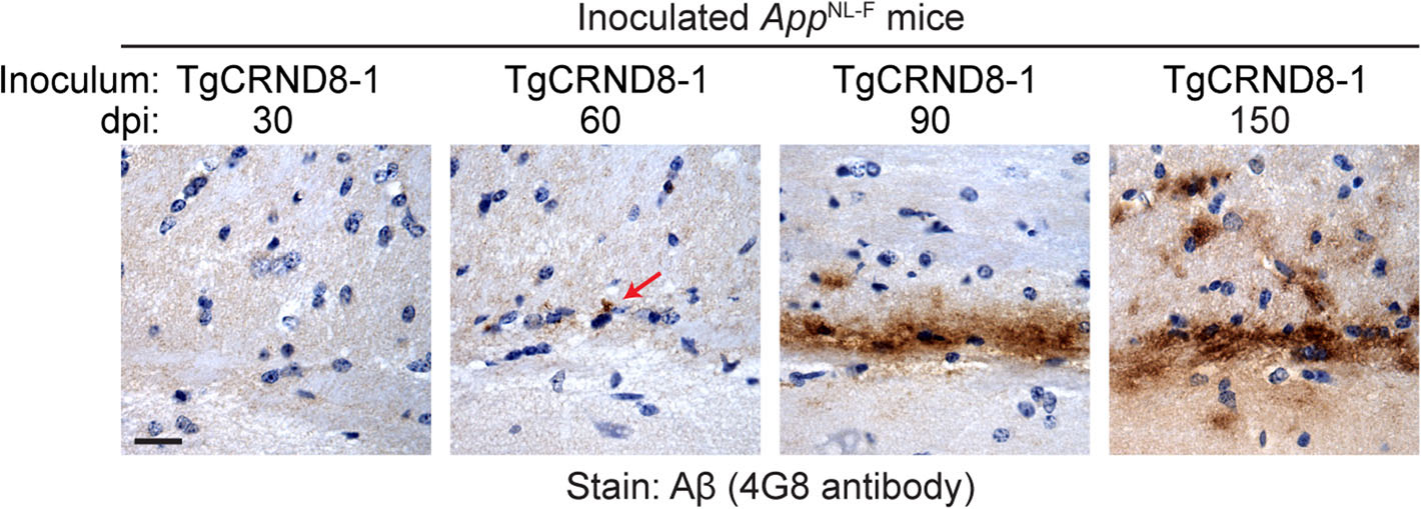
Time-course of induced Aβ deposition in the brains of Aβ-inoculated *App*^NL-F^ mice. Female mice were inoculated with partially purified Aβ aggregates from a TgCRND8 mouse (TgCRND8–1 sample) and brains were analyzed by Aβ immunohistochemistry (4G8 antibody) at the indicated dpi. Representative sections from the corpus callosum of inoculated mice are shown. Induced Aβ deposition was first apparent at 60 dpi (red arrow). Scale bar: 20 μm

To investigate the kinetics of induced Aβ deposition in *App*^NL-F^ mice, we injected groups of mice with partially purified Aβ aggregates from a TgCRND8 mouse and examined their brains by immunohistochemistry for Aβ pathology at 30, 60, 90, or 150 dpi. At 30 dpi, no induced Aβ deposits were observed anywhere within the brain (Fig. 2, Table 1), suggesting that the Aβ deposits observed at later timepoints are not due to the persistence of inoculated Aβ aggregates. Trace amounts of induced Aβ deposits within the subcallosal region were apparent at 60 dpi, and robust Aβ deposition was observed at both 90 and 150 dpi (Fig. 2, Table 1). The kinetics of the appearance of meningeal Aβ-CAA and Aβ deposits within the subcallosal region were similar, whereas Aβ deposits within the olfactory bulb appeared later and were not present in all Aβ-inoculated animals at 150 dpi (Table 1).

### Purification of brain-derived Aβ aggregates

**Fig. 3:**
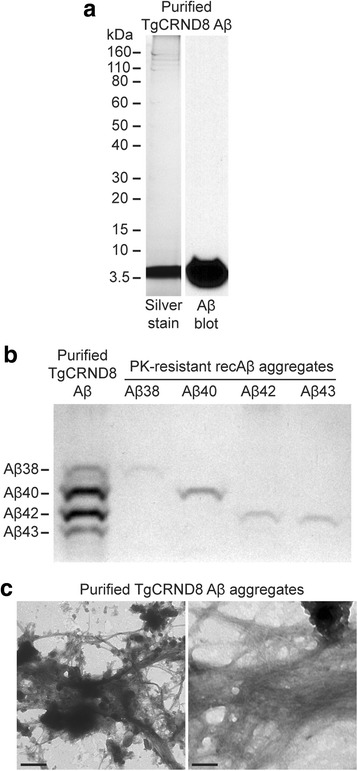
Purification of Aβ aggregates from the brain of an aged TgCRND8 mouse. **a** The purity of the isolated TgCRND8 Aβ aggregates was analysed by SDS-PAGE followed by either silver staining (left) or immunoblotting with the anti-Aβ antibody 6E10. **b** The Aβ isoform composition of the purified TgCRND8 Aβ aggregates was determined by separation on a Bicine/Tris 8 M urea gel followed by silver staining. PK-resistant Aβ fibrils prepared from various recombinant Aβ isoforms were used as controls. **c** Ultrastructural analysis of purified TgCRND8 Aβ aggregates by electron microscopy. Scale bars: 500 nm (left image) or 100 nm (right image)

To facilitate the further characterization of induced Aβ deposits in *App*^NL-F^ mice, we sought to maximize the extent of induced Aβ pathology. It has been previously reported that fully purified Aβ aggregates from the brains of TgCRND8 mice potently seed the deposition of Aβ when intracerebrally injected into susceptible transgenic mice [61]. Therefore, we purified Aβ aggregates from the brain of an aged TgCRND8 mouse using a protocol that employs iodixanol gradients and PK digestion [57, 61]. SDS-PAGE analysis followed by silver staining and/or immunoblotting revealed that Aβ was the main component of the purified material (Fig. 3a). For a more comprehensive analysis of the purified aggregates we employed a Bicine/Tris SDS-PAGE system containing urea that permits individual Aβ peptide isoforms to be resolved. Aβ42 and Aβ40 were the predominant peptides present in the purified aggregates, as expected for TgCRND8 mice, but appreciable amounts of Aβ38 and Aβ43 peptides were also detected (Fig. 3b). Using electron microscopy we confirmed that the purified material consisted of large, dense aggregates composed of Aβ fibrils bundled with amorphous non-fibrillar material, presumably remnants of brain matter (Fig. 3c), similar to what has been previously reported [61].

### Injection of purified Aβ aggregates into *App*^NL-F^ mice

Purified Aβ aggregates were intracerebrally inoculated into 6-week-old *App*^NL-F^ mice and then induction of cerebral Aβ deposition was assessed at 130 dpi, which corresponds to ~ 6 months of age. Since we did not observe a prominent sex effect on Aβ42 levels in mice injected with partially purified Aβ aggregates (Additional file 5: Figure S4), we decided to exclusively utilize male mice for these studies to minimize the occurrence of spontaneous Aβ deposits. As negative controls, mice were injected with inoculum diluent and analyzed at 132 dpi, and another cohort of mice was inoculated with purified Aβ aggregates and analyzed at 14 dpi. None of the inoculated mice exhibited any overt signs of neurological illness at the time of sacrifice, although one of the Aβ-injected animals was found dead in its cage at 117 dpi.

**Fig. 4:**
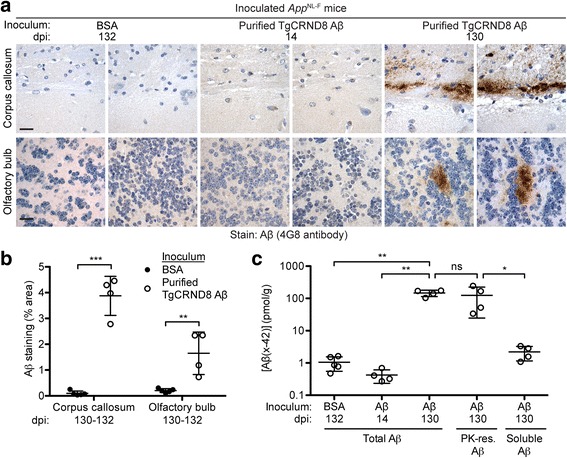
Induction of cerebral Aβ deposition in *App*^NL-F^ mice by inoculation with purified TgCRND8 Aβ aggregates. **a** Aβ immunohistochemistry with the 4G8 antibody on brain sections from male *App*^NL-F^ mice at 132 dpi with BSA, or at 14 or 130 dpi with purified TgCRND8 Aβ aggregates. Representative images of the corpus callosum and olfactory bulb from two independent mice per inoculation group are shown. Scale bars: 20 μm. **b** Quantification of Aβ staining (percentage area stained; mean ± s.d.) in the corpus callosum and olfactory bulb of *App*^NL-F^ mice inoculated with either BSA (n = 5) or purified TgCRND8 Aβ aggregates (*n* = 4) at 130–132 dpi. ***P < 0.001, **P < 0.01. **c** Total (formic acid-extractable), PK-resistant, and soluble (diethylamine-extractable) Aβ42 levels (mean ± s.d.) in brain homogenates from male *App*^NL-F^ mice at 130–132 dpi with either BSA (n = 5) or purified TgCRND8 Aβ aggregates (n = 4) as well as total Aβ42 levels from mice at 14 dpi with purified TgCRND8 Aβ aggregates (n = 4) were determined by ELISA. **P < 0.01; *P < 0.05; ns, not significant

Immunohistochemical analysis of ipsilateral brain sections from *App*^NL-F^ mice inoculated with purified TgCRND8 Aβ aggregates revealed the presence of induced Aβ deposits. Similar to what was observed in mice injected with partially purified Aβ aggregates, the induced deposits in mice injected with fully purified Aβ aggregates were mostly diffuse or granular in nature and were located primarily in the subcallosal region and olfactory bulb (Fig. 4a, b, Table 1). Unlike in mice injected with partially purified Aβ aggregates, 100% of mice inoculated with fully purified Aβ aggregates exhibited Aβ pathology within the olfactory bulb. None of the control-inoculated mice exhibited any Aβ deposits in their brains at 130 dpi, consistent with the paucity of Aβ deposits in the brains of uninoculated, 6-month-old male *App*^NL-F^ mice (Additional file 3: Table S1). Moreover, no Aβ deposits were observed in any of the mice at 14 dpi with purified Aβ aggregates, proving that the deposits observed at 130 dpi do not correspond to the injected material itself (Fig. 4a, Table 1).

Analysis of Aβ42 levels in brain homogenates prepared from the contralateral hemisphere of *App*^NL-F^ mice inoculated with purified TgCRND8 Aβ aggregates revealed that they accumulated ~ 100-fold more Aβ than control-inoculated mice (Fig. 4c). On average, the brains of mice that received purified TgCRND8 Aβ aggregates contained approximately twice the amount of Aβ42 than mice inoculated with partially purified TgCRND8 Aβ aggregates, despite being collected 20 days earlier. This is likely because these mice were injected with ~ 50-fold more Aβ aggregates than mice that received partially purified Aβ. At 14 dpi, Aβ42 levels in mice injected with purified Aβ aggregates were comparable to those in BSA-injected mice at 130 dpi, again indicating that the majority of the inoculated material is rapidly cleared from the brain and that amplification of the injected Aβ seeds occurred over the course of the experiment. Aβ42 levels in the brains of animals injected with purified Aβ aggregates were similar to those present in the brains of uninoculated *App*^NL-F^ mice at 12 months of age (Additional file 2: Figure S2a), indicating a significant acceleration of Aβ accumulation. Levels of PK-resistant Aβ42 in the brains of mice inoculated with purified Aβ aggregates were similar to total Aβ42 levels suggesting that the majority of the accumulated Aβ species were aggregates (Fig. 4c). Consistent with this notion, levels of soluble Aβ42 in mice injected with purified Aβ aggregates, as determined by diethylamine extraction of brain homogenates, were significantly lower than the levels of PK-resistant Aβ42 and were comparable to the levels of total Aβ42 present in control-inoculated animals (Fig. 4c). Taken together, these results demonstrate that robust cerebral Aβ deposition can be induced in *App*^NL-F^ mice by inoculation with purified Aβ aggregates.

### Characterization of induced Aβ deposits in mice inoculated with purified Aβ aggregates

**Fig. 5:**
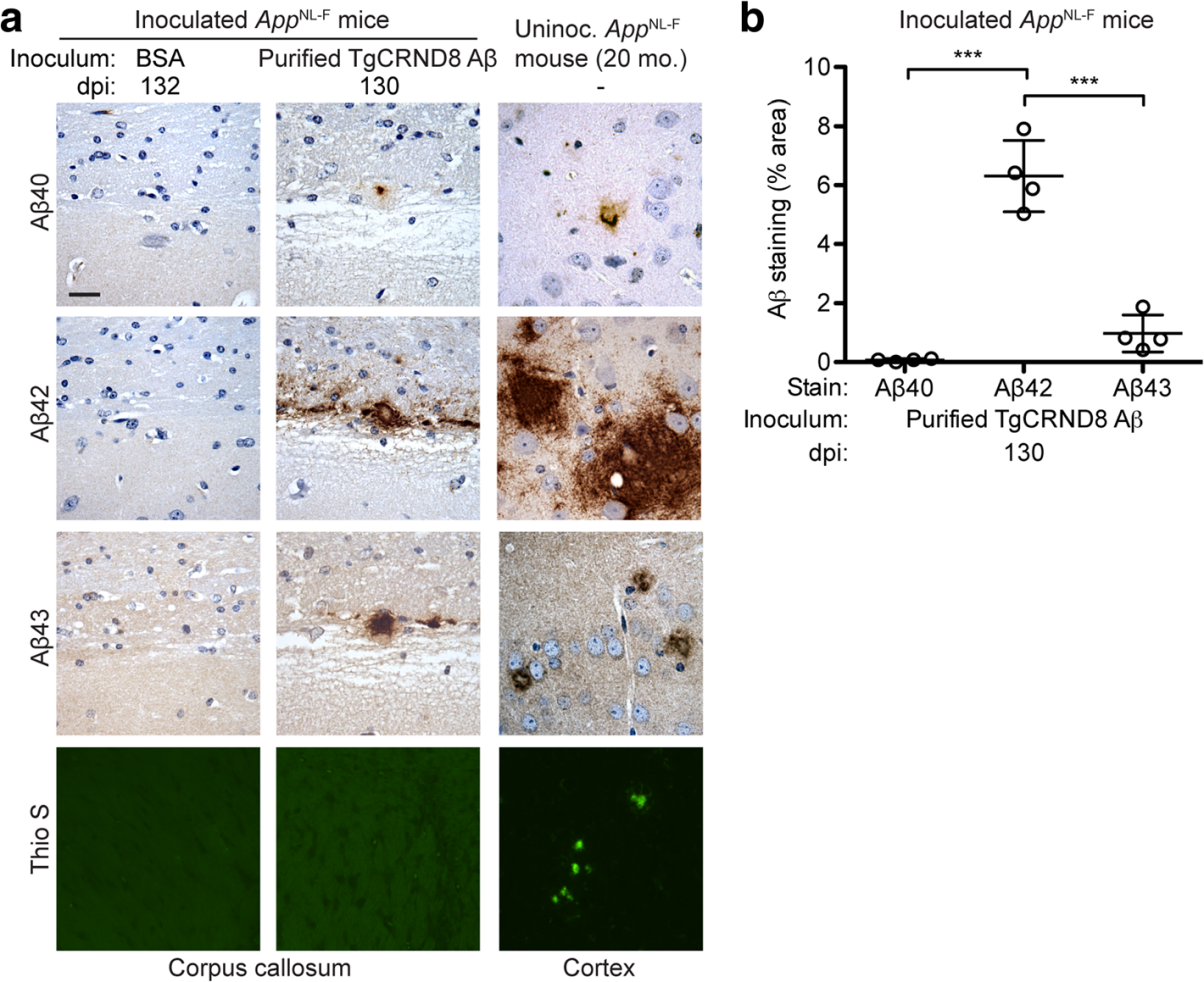
Characterization of induced Aβ deposits in *App*^NL-F^ mice inoculated with purified TgCRND8 Aβ aggregates. **a** Brain sections from male *App*^NL-F^ mice at 130–132 dpi with either BSA or purified TgCRND8 Aβ aggregates were analyzed by immunohistochemistry for Aβ40 (11A50-B10 antibody), Aβ42 (12F4 antibody), or Aβ43 (9C4 antibody), or by staining with Thio S. Brain sections from 20-month-old uninoculated male *App*^NL-F^ mice were used as a control. Representative images of the corpus callosum from inoculated mice and the cortex of uninoculated mice are shown. Cortical Aβ deposits were used for comparison since the Aβ pathology within the corpus callosum of aged, uninoculated *App*^NL-F^ mice is relatively mild compared to the robust Aβ deposition present in the cortex. Scale bar: 20 μm (applies to all images). **b** Quantification of Aβ40, Aβ42, and Aβ43 staining (percentage area stained; mean ± s.d.) in the corpus callosum of *App*^NL-F^ mice at 130 dpi with purified TgCRND8 Aβ aggregates (n = 4). ***P < 0.001

Immunohistochemical analysis of *App*^NL-F^ mice inoculated with purified TgCRND8 Aβ aggregates using antibodies against specific Aβ peptide isoforms showed that the induced Aβ aggregates in the subcallosal region were predominantly composed of Aβ42 (Fig. 5a, b). All of the mice injected with purified Aβ aggregates also exhibited some Aβ43 staining, particularly in areas with higher amounts of Aβ deposition (Fig. 5a), but the extent of staining was ~ 6-fold lower than for Aβ42. Aβ40 immunoreactivity within the induced deposits was either very weak or non-existent (Fig. 5a, b). No staining was observed with any of these antibodies in brain sections from buffer-injected animals (Fig. 5a). The relative abundance of Aβ isoforms within the induced Aβ deposits was similar to the composition of parenchymal Aβ plaques in the cortex of aged, uninoculated *App*^NL-F^ mice (Fig. 5a). Since high levels of oligomeric pyroglutamylated Aβ (pE3-Aβ) are observed in AD patients that possess the Iberian mutation [56], we also investigated levels of pE3-Aβ species in the brains of Aβ-inoculated *App*^NL-F^ mice. However, we observed no staining with the 9D5 antibody, which recognizes oligomeric pE3-Aβ [63], in either Aβ-inoculated mice or aged, uninoculated *App*^NL-F^ mice. Unexpectedly, the induced Aβ deposits in the subcallosal region of *App*^NL-F^ mice injected with purified Aβ aggregates were unreactive to Thioflavin S (Thio S), a dye that binds preferentially to repetitive β-sheet-rich structures present in amyloid aggregates (Fig. 5a). This suggests that the induced Aβ aggregates are distinct from the mature amyloid plaques observed in aged *App*^NL-F^ mice, many of which were reactive to Thio S (Fig. 5a).

### Injection of purified Aβ aggregates induces CAA in *App*^NL-F^ mice

**Fig. 6:**
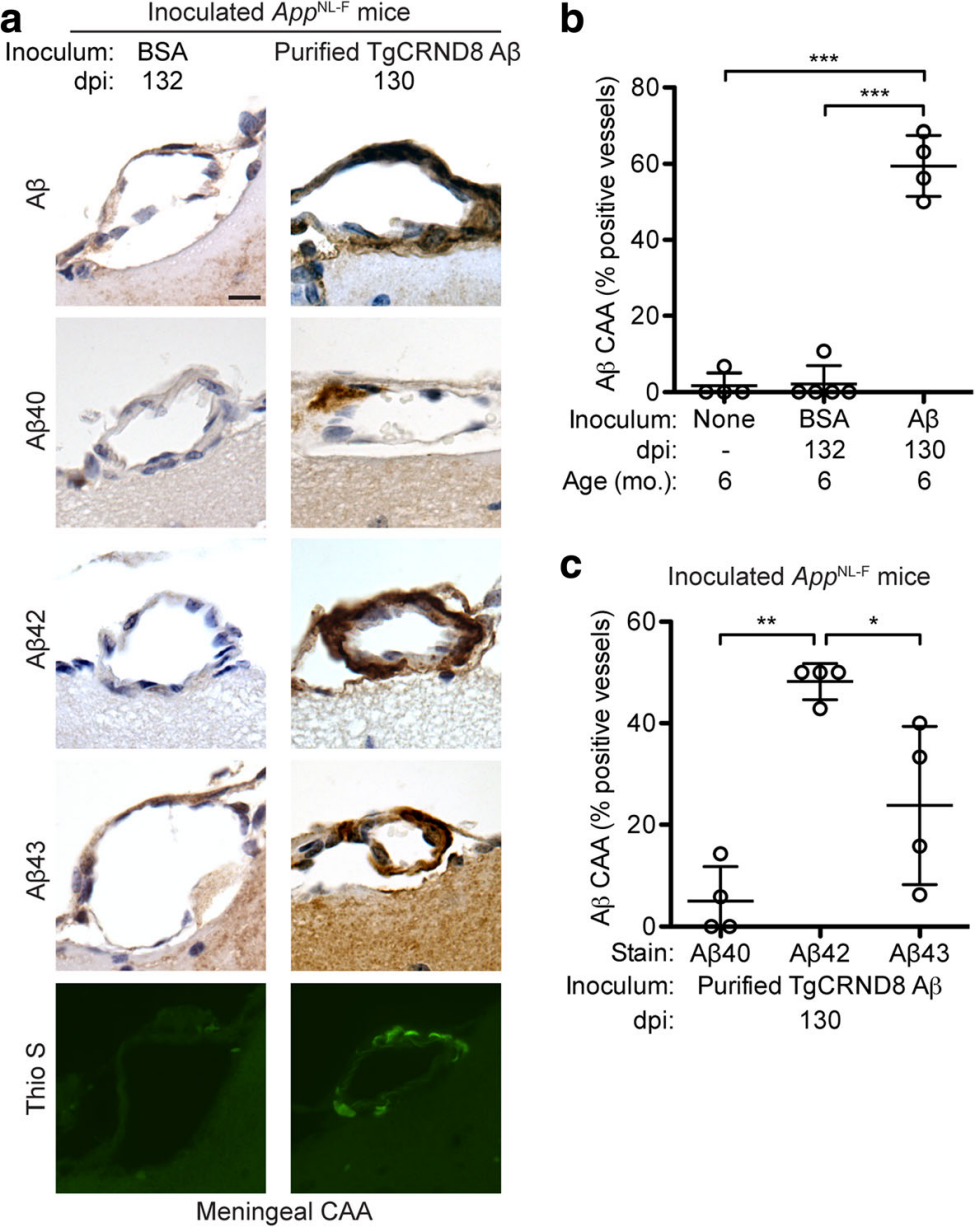
Aβ cerebral amyloid angiopathy in *App*^NL-F^ mice inoculated with purified TgCRND8 Aβ aggregates. **a** Brain sections from male *App*^NL-F^ mice at 130–132 dpi with either BSA or purified TgCRND8 Aβ aggregates were analyzed by immunohistochemistry for Aβ40 (11A50-B10 antibody), Aβ42 (12F4 antibody), or Aβ43 (9C4 antibody), or by staining with Thio S. Representative images of the meningeal blood vessels are shown. Scale bar: 10 μm (applies to all images). **b** Quantification of Aβ CAA (percentage of 4G8-positive vessels; mean ± s.d.) in the meningeal blood vessels of uninoculated *App*^NL-F^ mice at 6 months of age (n = 4) as well as *App*^NL-F^ mice at 130–132 dpi with either BSA (n = 5) or purified TgCRND8 Aβ aggregates (n = 4). ***P < 0.001. **c** Quantification of CAA Aβ isoform composition (percentage of Aβ40-, Aβ42-, or Aβ43-positive vessels; mean ± s.d.) in the meningeal blood vessels of *App*^NL-F^ mice at 130 dpi with purified TgCRND8 Aβ aggregates (n = 4). **P < 0.01, *P < 0.05

Consistent with what was observed in *App*^NL-F^ mice inoculated with partially purified Aβ aggregates (Fig. 1a), we found that inoculation of these mice with fully purified TgCRND8 Aβ aggregates also resulted in Aβ-CAA within meningeal blood vessels (Fig. 6a). The extent of meningeal Aβ-CAA was significantly higher in mice injected with purified Aβ aggregates compared to either BSA-injected or uninoculated control mice (Fig. 6b). The induced Aβ-CAA was largely restricted to the meningeal blood vessels on the outer surface of the brain and, like the induced Aβ deposits in the subcallosal region, was composed mainly of Aβ42 (Fig. 6c). Detectable amounts of Aβ40 and Aβ43 deposition within the meningeal blood vessels of Aβ-inoculated mice were also observed, although the extent of deposition tended to be much less than for Aβ42 (Fig. 6a, c). Unlike the induced Aβ deposits in the subcallosal region, induced Aβ CAA in mice that received fully purified Aβ aggregates was partially reactive to Thio S, indicative of the presence of Aβ amyloid (Fig. 6a). Thio S-positive blood vessels were observed in all (4/4) *App*^NL-F^ mice inoculated with purified Aβ aggregates but none (0/5) of the mice injected with BSA.

### Induction of Aβ deposition by injection with purified Aβ aggregates does not cause overt neuroinflammation in *App*^NL-F^ mice

The presence of neuroinflammation associated with Aβ deposits is frequently observed in AD and in transgenic mouse models of the disorder [29, 44]. Similarly, induced Aβ deposition in transgenic AD mouse models is often accompanied by reactive astrocytic gliosis [41, 61, 69]. Since increased staining for glial fibrillary acidic protein (GFAP), a marker for reactive astrocytes, and ionized calcium-binding adaptor molecule 1 (Iba1), a marker for activated microglia, was observed in the vicinity of Aβ plaques in the brains of aged *App*^NL-F^ mice [51], we checked whether the induced Aβ deposits in mice inoculated with purified Aβ aggregates were also associated with these markers of neuroinflammation. However, there were no significant differences in the extent of GFAP or Iba1 staining within the corpus callosum or the olfactory bulb of Aβ- or control-inoculated *App*^NL-F^ mice (Additional file 6: Figure S5). These results suggest that, at least at the specific timepoint analyzed (130 dpi), the induction of cerebral Aβ deposition in *App*^NL-F^ mice is not accompanied by significant neuroinflammation.

## Discussion

Here we investigated whether cerebral Aβ deposition can be induced in a non-APP-overexpressing AD mouse model by intracerebral inoculation with pre-formed Aβ aggregates. It has recently been suggested that many phenotypes ascribed to toxic Aβ assemblies in transgenic mouse models of AD, such as enhanced proteolysis of p35 to p25 by calpain, may actually be due to overexpression of APP [52]. However, similar to studies in APP-overexpressing transgenic mice or rats [22, 35, 41, 43, 50, 61], we observed robust induction of cerebral Aβ deposition upon inoculation of *App*^NL-F^ knock-in mice with either partially or fully purified, brain-derived Aβ aggregates, confirming that overexpression of APP is not a prerequisite for the prion-like propagation of Aβ aggregation. Thus, the prion-like propagation of Aβ aggregates is a physiologically plausible mechanism that may, at least in part, explain the observed spread of Aβ deposits within the brains of AD patients [62] and transgenic mouse models of the disease [25].

The presence of induced Aβ deposition in the subcallosal region of Aβ-injected *App*^NL-F^ mice was not unexpected since it seems to be a pathological signature of prion-like Aβ aggregate propagation following intracerebral inoculation [14, 60, 61, 69]. Accumulation of Aβ seeds and thus the presence of Aβ deposits within this region may be a by-product of the inoculation technique employed, which tends to deliver the inoculum to subcallosal regions on both sides of the brain [27, 64]. Aβ deposits were also observed in the corpus callosum of older *App*^NL-F^ mice, but this pathology was mild in comparison to the robust cortical Aβ pathology present at these ages. In contrast, cortical Aβ deposits were not observed in mice injected with Aβ aggregates. The reason for this inconsistency remains enigmatic, but this observation indicates that the patterns of induced and spontaneous Aβ deposition in *App*^NL-F^ mice are distinct. As mentioned above, a simple explanation could be that the inoculation technique preferentially delivers Aβ seeds to specific regions of the brain. The presence of induced Aβ aggregates in regions far distal to the inoculation site, such as the olfactory bulb, potentially suggests that spreading of Aβ aggregates has occurred. However, because we utilized a non-stereotactic injection technique, we cannot rule out that the inoculum itself reached the olfactory bulb, possibly via transportation within the brain vasculature. This possibility is supported by the observation that Aβ deposition within meningeal blood vessels was an early and robust phenotype in all Aβ-injected mice.

Like all AD mouse models involving expression of mutant APP [44], *App*^NL-F^ mice develop cerebral Aβ deposits but do not develop tau deposits or neurofibrillary tangles. Based on our preliminary analysis, levels of phosphorylated tau pathology were not increased in Aβ-inoculated *App*^NL-F^ mice compared to buffer-inoculated animals. Thus, Aβ-inoculated *App*^NL-F^ mice are not a suitable system for studying the effects of Aβ propagation on downstream pathological aspects of AD, such as tau polymerization and neuronal loss. The presence of the Iberian APP mutation in *App*^NL-F^ mice skews the distribution of Aβ peptide isoforms towards Aβ42, whereas Aβ40 is the more abundantly produced Aβ isoform in normal human brain. This could affect the propagation and deposition of Aβ, especially during early disease phases. *App*^NL/NL^ knock-in mice [51], which express APP containing only the Swedish mutation and thus produce the normal complement of Aβ peptide isoforms, would not have this issue but the induction of Aβ deposition may be prohibitively slow in this model.

The induced Aβ deposits in *App*^NL-F^ mice inoculated with purified TgCRND8 Aβ aggregates were similar to those observed in Aβ-inoculated APP-overexpressing lines such as APP23 and Tg2576 in that the deposits were primarily diffuse or granular in nature [35, 41]. However, unlike in previous reports [41, 61], we were unable to find any Thio S-positive induced parenchymal Aβ deposits in the Aβ-injected *App*^NL-F^ mice, suggesting that the induced aggregates were not composed of Aβ amyloids, despite the fact that abundant levels of protease-resistant Aβ were present. The reason for this discrepancy is unclear, but is not due to the inoculum used since purified TgCRND8 Aβ aggregates induced Thio S-positive Aβ deposits in the corpus callosum of APP23 mice [61]. The most likely explanation is that a longer incubation period following inoculation of Aβ aggregates into *App*^NL-F^ mice is required to obtain plaque-like aggregates. Unfortunately, the spontaneous appearance of plaques in *App*^NL-F^ mice beginning at ~ 6 months of age [51] precludes us from investigating prolonged timepoints in this model. It is also conceivable that elevated levels of APP expression in transgenic AD models may promote the development of plaque-like, Thio S-positive Aβ deposits, possibly via acting as a scaffold upon which Aβ aggregates can assemble. The lack of neuroinflammatory markers in Aβ-injected *App*^NL-F^ mice is likely related to the absence of plaque-like Aβ deposits, since elevated GFAP staining was associated with brain regions containing Thio S-positive induced Aβ deposits in APP23 mice [41, 61]. Collectively, our results are consistent with the induced Aβ deposits in *App*^NL-F^ mice being composed of smaller Aβ aggregates, which may exhibit an enhanced ability to propagate within the brain.

The induction of cerebral Aβ deposition has also been observed in other systems without APP overexpression. Aβ deposits (senile plaques and CAA) were found in the brains of marmosets that had been injected with AD brain extract 6–7 years earlier [2, 3, 48], revealing that Aβ deposition can be induced in a wild-type (non-genetically-modified) animal. However, the marmoset paradigm is not ideal for studying Aβ propagation owing to the extremely long incubation periods required. A prion-like transmission mechanism has also been proposed to explain the presence of cerebral Aβ deposits and Aβ-CAA in humans that underwent neurosurgery or were exposed to contaminated growth hormone extracts or dura mater grafts [10, 16, 20, 23, 32, 33, 49], although the clinical relevance of these findings has not yet been fully elucidated [30, 31]. Even though we have found that cerebral Aβ deposition can be induced in mice that do not overexpress APP, the likelihood of cases of AD occurring due to exposure to exogenous Aβ aggregates, similar to what occasionally occurs in prion disease [8], is improbable. Previous studies have shown that brain extracts from either AD patients or aged Tg mice with abundant deposits of human Aβ peptide fail to induce cerebral Aβ deposition in non-transgenic mice [35, 41] or rats [50]. One potential explanation is that a humanized Aβ sequence is required for the efficient prion-like propagation of human Aβ aggregates. In prion disease, even a single amino acid difference between the inoculated prions and the prion protein sequence of the host can significantly impede disease transmission [4]. Since the sequences of rodent and human Aβ differ at three positions, a “transmission barrier” may limit the induction of mouse Aβ aggregates by human Aβ seeds. However, this putative barrier clearly does not extend to non-Aβ regions of APP, since we observed robust induction of Aβ deposition in *App*^NL-F^ mice, which express murine APP but produce humanized Aβ.

While our results demonstrate that APP overexpression is not required for the induction of cerebral Aβ deposition, we cannot rule out a role for APP expressed at physiological levels in the propagation of Aβ aggregates. APP may act as a receptor for Aβ [36, 40, 55, 58], potentially facilitating the assembly and/or spread of Aβ aggregates. Ascribing a role for APP in this process may be difficult because Aβ generation, which is required for the propagation of Aβ aggregates, is intrinsically linked to APP expression. It may be feasible to answer this question by performing Aβ seeding experiments in transgenic mice that produce either secreted or membrane-anchored forms of Aβ without requiring excision from APP [45], following backcross to a murine *App* null background. Aβ aggregate seeds capable of inducing cerebral Aβ deposition in APP23 mice persist in the brains of *App* knockout mice for up to 6 months post-inoculation [71], suggesting that other, non-APP receptors capable of trapping Aβ aggregates may exist in the brain. Candidates include the cellular prion protein [39] and PirB [37]. Other small neuropeptides, such as somatostatin [67], may also influence the aggregation and propagation of Aβ.

## Conclusions

The concept of self-propagating, prion-like protein aggregates and their potential role in the pathogenesis of neurodegenerative diseases remains controversial [66]. For example, another potential explanation for the apparent spread of protein aggregates is that different regions within the brain may exhibit selective vulnerability to Aβ deposition [66], a process that may be regulated by neuronal activity [6]. Furthermore, the nomenclature of these prion-like protein aggregates has been hotly debated [1, 24, 46, 65]. Our finding that the induction and spreading of Aβ aggregates can be achieved in mice that do not over-express APP adds to the growing literature on the prion-like propagation of Aβ aggregation and the quest to understand its relevance for AD and Aβ-CAA.

## Additional files

Additional file 1: **Figure S1.** Analysis of APP levels in uninoculated *App*^NL-F^ mice. a Immunoblots of brain homogenates from uninoculated wild-type (C57Bl/6) and *App*^NL-F^ mice probed with antibodies that recognize both human (Hu) and mouse (Mo) APP (22C11; left blot) or just human APP (6E10; right blot). Blots were re-probed with an actin antibody. b Quantification of APP levels (mean ± s.d.; normalized to actin levels) in brain homogenates from wild-type and *App*^NL-F^ mice (*n* = 3) each. APP levels were not significantly different between the two groups (*P* = 0.30), as assessed by a two-tailed, unpaired *t*-test. (TIFF 272 kb)

Additional file 2: **Figure S2.** Kinetics of spontaneous Aβ deposition in the brains of uninoculated *App*^NL-F^ mice. a Formic acid-extractable Aβ42 levels (mean ± s.d.) in brain homogenates from male *App*^NL-F^ mice at the indicated ages (n = 3 for each age) were determined by ELISA. b Immunoblot of insoluble, PK-resistant Aβ species in brain homogenates from two distinct male *App*^NL-F^ mice for each of the indicated ages. Aβ was detected using the antibody 6E10. **c** Brain sections from either male (M) or female (F) *App*^NL-F^ mice at the indicated ages were subjected to immunohistochemistry with the 4G8 antibody, which recognizes Aβ. Representative images from the cortex, olfactory bulb, and the meningeal blood vessels are shown. Scale bars: 20 μm (cortex and olfactory bulb images) or 10 μm (meningeal CAA images). (TIFF 6186 kb)

Additional file 3: **Table S1.** Summary of Aβ pathology in uninoculated *App*^NL-F^ mice. (DOCX 14 kb)

Additional file 4: **Figure S3.** Callosal Aβ pathology in uninoculated and Aβ-inoculated *App*^NL-F^ mice. Brain sections from male *App*^NL-F^ mice at the indicated ages that were either injected with partially purified Aβ aggregates (TgCRND8–1 sample) or left uninoculated were subjected to Aβ immunohistochemistry using the 4G8 antibody. In Aβ-inoculated mice, the induced Aβ deposits were found within the subcallosal region whereas in uninoculated mice the Aβ deposits were present within the corpus callosum itself. Scale bars: 20 μm. (TIFF 757 kb)

Additional file 5: **Figure S4** Sex does not influence Aβ42 levels in Aβ-inoculated *App*^NL-F^ mice. Levels of formic acid-extractable Aβ42 (mean ± s.d.), as determined by ELISA, in brain homogenates prepared from male (*n* = 14) or female (*n* = 15) *App*^NL-F^ mice that had been inoculated with partially purified Aβ aggregates (150–152 dpi) are not significantly different from each other (*P* = 0.066 by a two-tailed, unpaired *t*-test). Data was pooled from inoculation experiments involving the TgCRND8–1 (black circles), TgCRND8–2 (red circles), AD-1 (blue circles), and AD-2 (green circles) samples. (TIFF 135 kb)

Additional file 6: **Figure S5.** Induced Aβ deposits in *App*^NL-F^ mice inoculated with purified TgCRND8 Aβ aggregates are not associated with increased levels of astrocytic gliosis or microglial activation. a Brain sections from male *App*^NL-F^ mice injected with either BSA or purified TgCRND8 Aβ aggregates (130–132 dpi) were analyzed by immunohistochemistry for GFAP or Iba1. Representative images of the corpus callosum and olfactory bulb are shown. Scale bar: 20 μm (applies to all images). b-c Quantification of GFAP (b) and Iba1 (c) staining (percentage area stained; mean ± s.d.) in the corpus callosum and olfactory bulb of *App*^NL-F^ mice inoculated with either BSA (*n* = 5) or purified TgCRND8 Aβ aggregates (*n* = 4). ns, not significant (*P* > 0.05). (TIFF 3135 kb)

## Abbreviations

AD: Alzheimer’s disease
APP: Amyloid precursor protein
Aβ: β-amyloid peptide
BSA: Bovine serum albumin
CAA: Cerebral amyloid angiopathy
dpi: Days post-inoculation
GFAP: Glial fibrillary acidic protein
Iba1: Ionized calcium-binding adaptor molecule 1
PK: Proteinase K
PTA: Phosphotungstic acid
Tg: Transgenic
Thio S: Thioflavin S
