# Supplementary figures and images for: Prion-like propagation of β-amyloid aggregates in the absence of APP overexpression
Source: Acta Neuropathol Commun. 2018 Apr 3;6:26. doi: 10.1186/s40478-018-0529-x (PMC5883524; doi:10.1186/s40478-018-0529-x)

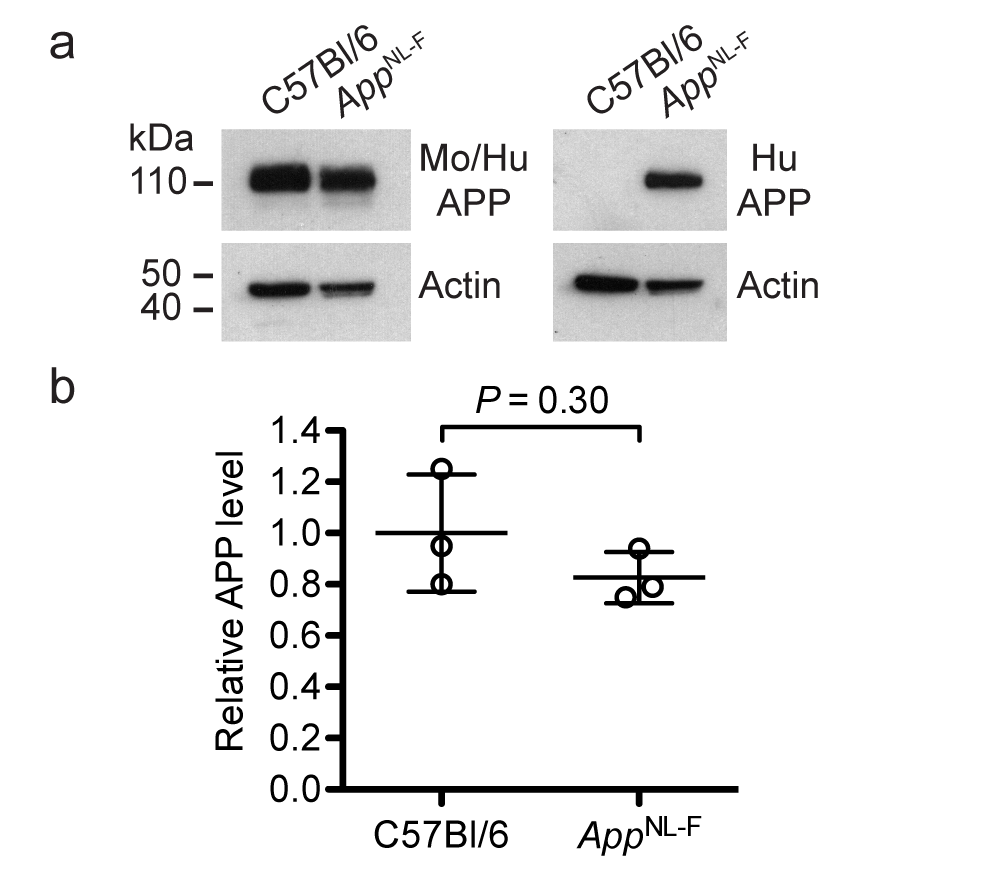

Supplement: Supplementary file 1 — Figure S1. Analysis of APP levels in uninoculated AppNL-F mice. a Immunoblots of brain homogenates from uninoculated wild-type (C57Bl/6) and AppNL-F mice probed with antibodies that recognize both human (Hu) and mouse (Mo) APP (22C11; left blot) or just human APP (6E10; right blot). Blots were re-probed with an actin antibody. b Quantification of APP levels (mean ± s.d.; normalized to actin levels) in brain homogenates from wild-type and AppNL-F mice (n = 3) each. APP levels were not significantly different between the two groups (P = 0.30), as assessed by a two-tailed, unpaired t-test. (TIFF 272 kb) [file 40478_2018_529_MOESM1_ESM.tif]

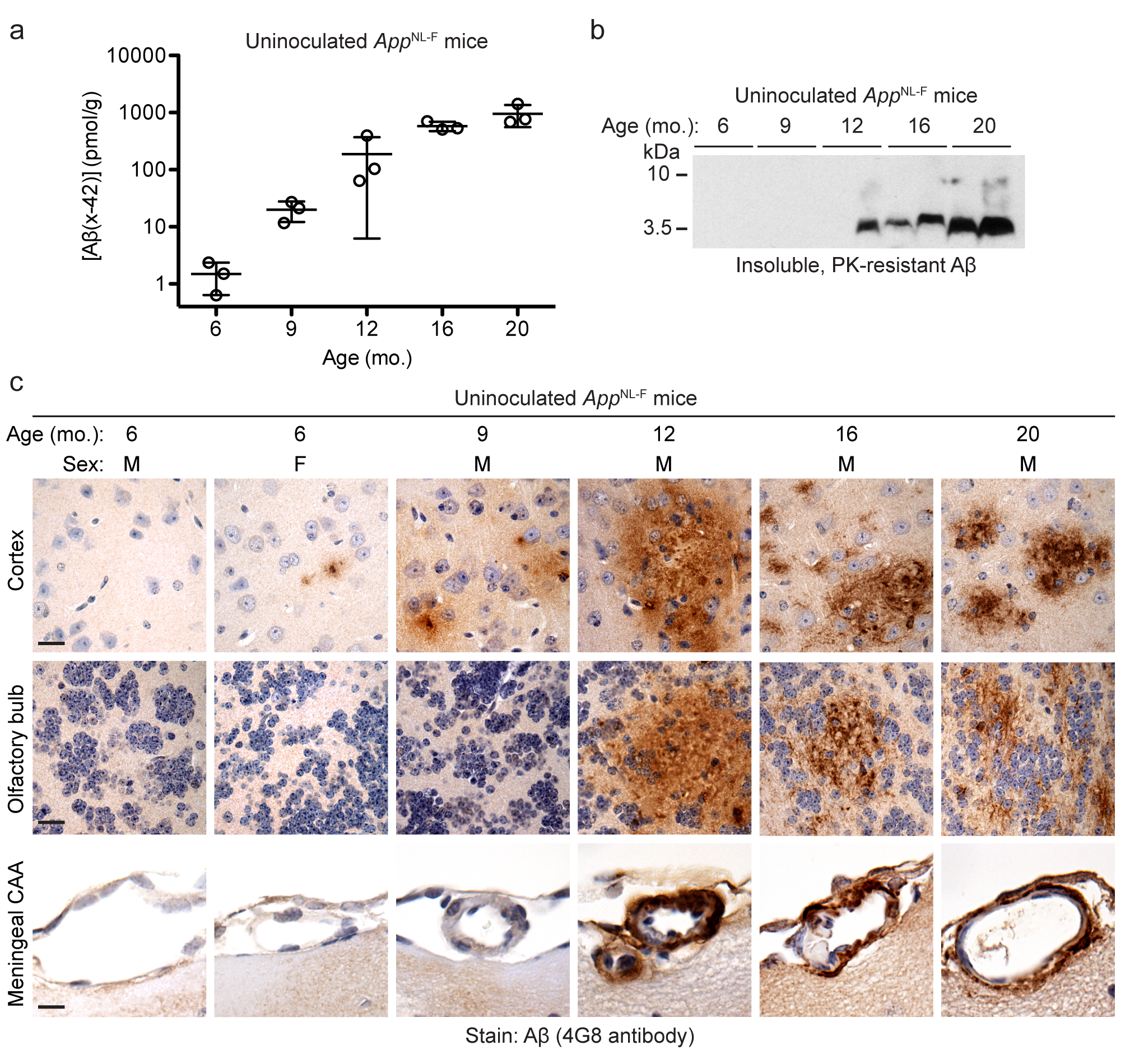

Supplement: Supplementary file 2 — Figure S2. Kinetics of spontaneous Aβ deposition in the brains of uninoculated AppNL-F mice. a Formic acid-extractable Aβ42 levels (mean ± s.d.) in brain homogenates from male AppNL-F mice at the indicated ages (n = 3 for each age) were determined by ELISA. b Immunoblot of insoluble, PK-resistant Aβ species in brain homogenates from two distinct male AppNL-F mice for each of the indicated ages. Aβ was detected using the antibody 6E10. c Brain sections from either male (M) or female (F) AppNL-F mice at the indicated ages were subjected to immunohistochemistry with the 4G8 antibody, which recognizes Aβ. Representative images from the cortex, olfactory bulb, and the meningeal blood vessels are shown. Scale bars: 20 μm (cortex and olfactory bulb images) or 10 μm (meningeal CAA images). (TIFF 6186 kb) [file 40478_2018_529_MOESM2_ESM.tif]

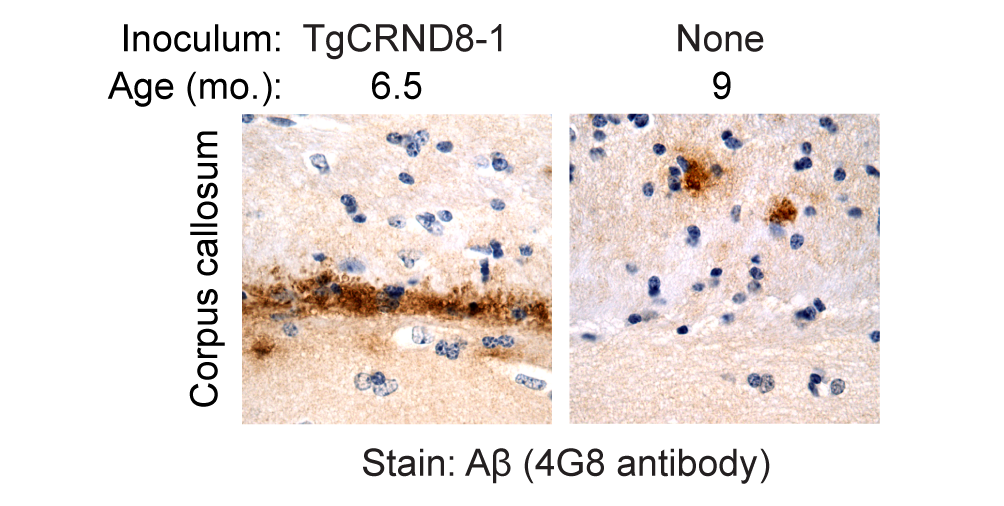

Supplement: Supplementary file 4 — Figure S3. Callosal Aβ pathology in uninoculated and Aβ-inoculated AppNL-F mice. Brain sections from male AppNL-F mice at the indicated ages that were either injected with partially purified Aβ aggregates (TgCRND8–1 sample) or left uninoculated were subjected to Aβ immunohistochemistry using the 4G8 antibody. In Aβ-inoculated mice, the induced Aβ deposits were found within the subcallosal region whereas in uninoculated mice the Aβ deposits were present within the corpus callosum itself. Scale bars: 20 μm. (TIFF 757 kb) [file 40478_2018_529_MOESM4_ESM.tif]

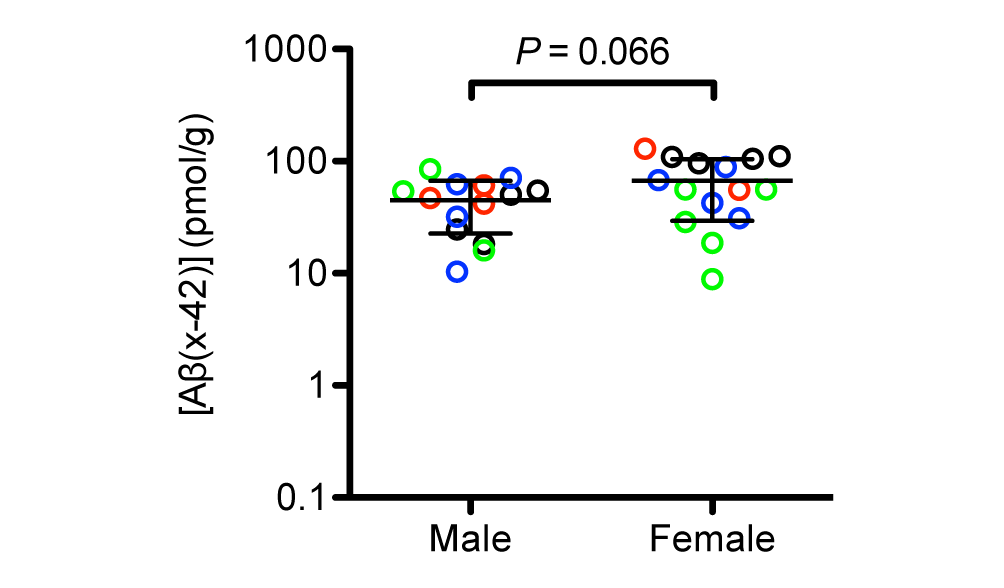

Supplement: Supplementary file 5 — Figure S4 Sex does not influence Aβ42 levels in Aβ-inoculated AppNL-F mice. Levels of formic acid-extractable Aβ42 (mean ± s.d.), as determined by ELISA, in brain homogenates prepared from male (n = 14) or female (n = 15) AppNL-F mice that had been inoculated with partially purified Aβ aggregates (150–152 dpi) are not significantly different from each other (P = 0.066 by a two-tailed, unpaired t-test). Data was pooled from inoculation experiments involving the TgCRND8–1 (black circles), TgCRND8–2 (red circles), AD-1 (blue circles), and AD-2 (green circles) samples. (TIFF 135 kb) [file 40478_2018_529_MOESM5_ESM.tif]

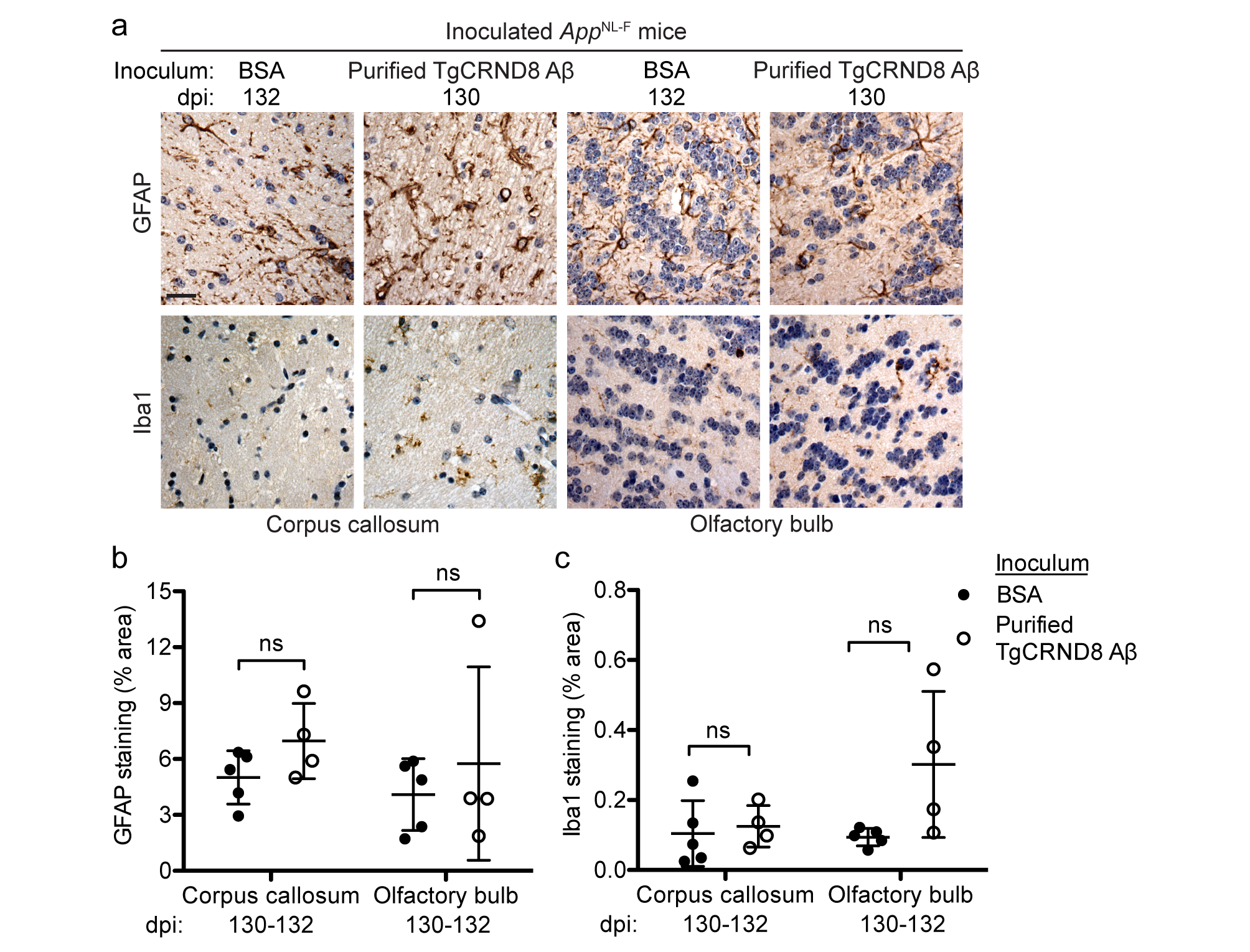

Supplement: Supplementary file 6 — Figure S5. Induced Aβ deposits in AppNL-F mice inoculated with purified TgCRND8 Aβ aggregates are not associated with increased levels of astrocytic gliosis or microglial activation. a Brain sections from male AppNL-F mice injected with either BSA or purified TgCRND8 Aβ aggregates (130–132 dpi) were analyzed by immunohistochemistry for GFAP or Iba1. Representative images of the corpus callosum and olfactory bulb are shown. Scale bar: 20 μm (applies to all images). b-c Quantification of GFAP (b) and Iba1 (c) staining (percentage area stained; mean ± s.d.) in the corpus callosum and olfactory bulb of AppNL-F mice inoculated with either BSA (n = 5) or purified TgCRND8 Aβ aggregates (n = 4). ns, not significant (P > 0.05). (TIFF 3135 kb) [file 40478_2018_529_MOESM6_ESM.tif]
