# Supplementary material for: Prion-like propagation of β-amyloid aggregates in the absence of APP overexpression
Source: Acta Neuropathol Commun. 2018 Apr 3;6:26. doi: 10.1186/s40478-018-0529-x (PMC5883524; doi:10.1186/s40478-018-0529-x)
Supplement: Supplementary file 3 — Table S1. Summary of Aβ pathology in uninoculated AppNL-F mice. (DOCX 14 kb) [file 40478_2018_529_MOESM3_ESM.docx]

Table S1

| **Age (mo.)** | **Sex** | **Aβ deposition^1^ (n/n_0_^2^)** | | | | |
| --- | --- | --- | --- | --- | --- | --- |
|  |  | **Corpus callosum** | **Hippocampus** | **Cortex** | **Olfactory bulb** | **Meningeal CAA** |
| 6 | Male | 1/4 | 0/4 | 1/4 | 0/4 | 1/4 |
|  | Female | 0/4 | 0/4 | 4/4 | 0/4 | 0/4 |
| 9 | Male | 4/4 | 0/4 | 4/4 | 0/4 | 1/4 |
|  | Female | 4/5 | 0/5 | 5/5 | 0/5 | 1/5 |
| 12 | Male | 2/2 | 1/2 | 2/2 | 2/2 | 2/2 |
|  | Female | 3/3 | 1/3 | 3/3 | 3/3 | 2/3 |
| 16 | Male | 3/3 | 3/3 | 4/4 | 3/3 | 4/4 |
|  | Female | 5/5 | 5/5 | 5/5 | 5/5 | 5/5 |
| 20 | Male | 4/4 | 4/4 | 4/4 | 4/4 | 4/4 |
|  | Female | 3/3 | 3/3 | 3/3 | 3/3 | 3/3 |

^1^Aβ deposition was assessed by immunohistochemistry using the anti-Aβ antibody 4G8

^2^n, number of positive mice; n_0_, number of analyzed mice; a sample was scored as positive if there was clear evidence for Aβ deposition or at least one Aβ CAA-positive meningeal blood vessel
